# The role of inflammasomes in vascular cognitive impairment

**DOI:** 10.1186/s13024-021-00506-8

**Published:** 2022-01-09

**Authors:** Luting Poh, Wei Liang Sim, Dong-Gyu Jo, Quynh Nhu Dinh, Grant R. Drummond, Christopher G. Sobey, Christopher Li-Hsian Chen, Mitchell K. P. Lai, David Y. Fann, Thiruma V. Arumugam

**Affiliations:** 1 Department of Physiology, Yong Loo Lin School of Medicine, National University of Singapore, Singapore, Singapore; 2 School of Pharmacy, Sungkyunkwan University, Suwon, Republic of Korea; 3 Centre for Cardiovascular Biology and Disease Research, Department of Physiology, Anatomy and Microbiology, La Trobe University, Bundoora, VIC Australia; 4 Memory Aging and Cognition Centre, Department of Pharmacology, Yong Loo Lin School of Medicine, National University of Singapore, Singapore, Singapore; 5 Department of Psychological Medicine, Yong Loo Lin School of Medicine, National University of Singapore, Singapore, Singapore; 6 Department of Biochemistry, Yong Loo Lin School of Medicine, National University of Singapore, Singapore, Singapore; 7 Healthy Longevity Translational Research Program, Yong Loo Lin School of Medicine, National University of Singapore, Singapore, Singapore; 8 Centre for Healthy Longevity, National University Health System (NUHS), Singapore, Singapore

**Keywords:** Inflammasome, Inflammation, Chronic cerebral Hypoperfusion, Vascular cognitive impairment, Vascular dementia

## Abstract

There is an increasing prevalence of Vascular Cognitive Impairment (VCI) worldwide, and several studies have suggested that Chronic Cerebral Hypoperfusion (CCH) plays a critical role in disease onset and progression. However, there is a limited understanding of the underlying pathophysiology of VCI, especially in relation to CCH. Neuroinflammation is a significant contributor in the progression of VCI as increased systemic levels of the proinflammatory cytokine interleukin-1β (IL-1β) has been extensively reported in VCI patients. Recently it has been established that CCH can activate the inflammasome signaling pathways, involving NLRP3 and AIM2 inflammasomes that critically regulate IL-1β production. Given that neuroinflammation is an early event in VCI, it is important that we understand its molecular and cellular mechanisms to enable development of disease-modifying treatments to reduce the structural brain damage and cognitive deficits that are observed clinically in the elderly. Hence, this review aims to provide a comprehensive insight into the molecular and cellular mechanisms involved in the pathogenesis of CCH-induced inflammasome signaling in VCI.

## Dementia: a focus on vascular cognitive impairment

Dementia describes a set of symptoms that occur when the brain is damaged by injury or disease. These commonly include progressive deterioration in memory, thinking and behavior, and ultimately the ability to perform everyday activities [1]. Dementia can be caused by many neurological disorders including Alzheimer’s disease (AD), frontotemporal dementia, Lewy body dementia and vascular dementia (VaD). An estimated 35.6 million people worldwide were diagnosed with dementia in 2010, with these numbers expected to double every 20 years [2]. In particular, the proportion of VaD patients among the entire dementia population is reported to be approximately 15–20% in North America and Europe [3, 4], and 30% in Asia and developing countries [5–7].

In recent decades, increasing evidence has shown vascular diseases contributing to cognitive impairment and memory deficits, with an underlying vascular component in the etiology of most forms of dementia [8, 9]. Several studies have shown that with the inclusion of dementia arising from mixed neuropathologies, the percentage of the demented population with a contributing vascular cause may be up to 70% [10, 11]. To effectively target cognitive impairment due to vascular injury or diseases, the term Vascular Cognitive Impairment (VCI) was introduced to encapsulate the whole spectrum of disease, ranging from subjective cognitive impairment, mild cognitive impairment to dementia associated with underlying cerebrovascular disease burden [12, 13].

VCI arises from heterogeneous cerebrovascular pathologies with diverse vascular etiologies. From the perspective of vascular injury, the origin and type of vascular occlusion, hemorrhage, distribution of arterial territories, and vessel size are the common causes of the types of vascular pathologies due to large vessel diseases, small vessel diseases, ischemic-hypoperfusive and hemorrhagic diseases [14]. The underlying vascular etiologies for VCI have been extensively reviewed in the statement released by the International Society for Vascular Behavioral and Cognitive Disorders (VASCOG) [15].

## Chronic Cerebral Hypoperfusion (CCH): a primary driver of VCI

Pre-clinical vascular diseases are difficult to detect until vascular lesions are formed and affect cognitive functions [16]. While numerous lines of evidence have identified risk factors associated with pre-clinical vascular diseases and VCI, the mechanisms by which these risk factors contribute to VCI pathologies resulting in cognitive impairment remain to be fully established [17–19]. Emerging evidence suggests that chronic cerebral hypoperfusion (CCH), as a result of vascular disease, could play a critical role in the pathophysiology of VCI [20–22]. CCH refers to a condition whereby cerebral blood flow (CBF) supply to the brain is reduced by 20 to 40% over a prolonged period. It can occur either to the whole brain or within specific brain regions [23, 24]. CCH is involved in the development of VCI as it is closely-associated with a number of major physiological vascular risk factors, VCI pathologies and cognitive decline [20, 25, 26]. It is well established CCH precedes white matter lesion (WML) formation and the presence of cerebral hypoperfusion together with white matter lesions, further exacerbates the decline in executive function and memory in VCI patients with dementia [27, 28].

Understanding the effect of CCH on cerebral function may explain its role in VCI. A continuous CBF plays an essential role in maintaining the brain’s structural and functional integrity [29]. At the neurovascular level, continuous blood supply to the brain parenchyma is necessary for essential functions such as neuronal activity, blood-brain barrier function and immune cell surveillance [8]. Disruption of blood flow to the brain is associated with a number of neurovascular dysfunctions such as endothelial dysfunction, glial activation, demyelination and blood-brain barrier breakdown as observed in VCI patients [30–32]. These observations warrant mechanistic investigations of VCI pathogenesis from the perspective of CCH. Various animal models have been developed to mimic a state of cerebral hypoperfusion as observed in VCI patients [33, 34]. By experimentally inducing CCH, these animal models provide a useful platform for understanding the underlying pathophysiology of VCI.

### Pathogenic molecular mechanisms of CCH

**Fig. 1:**
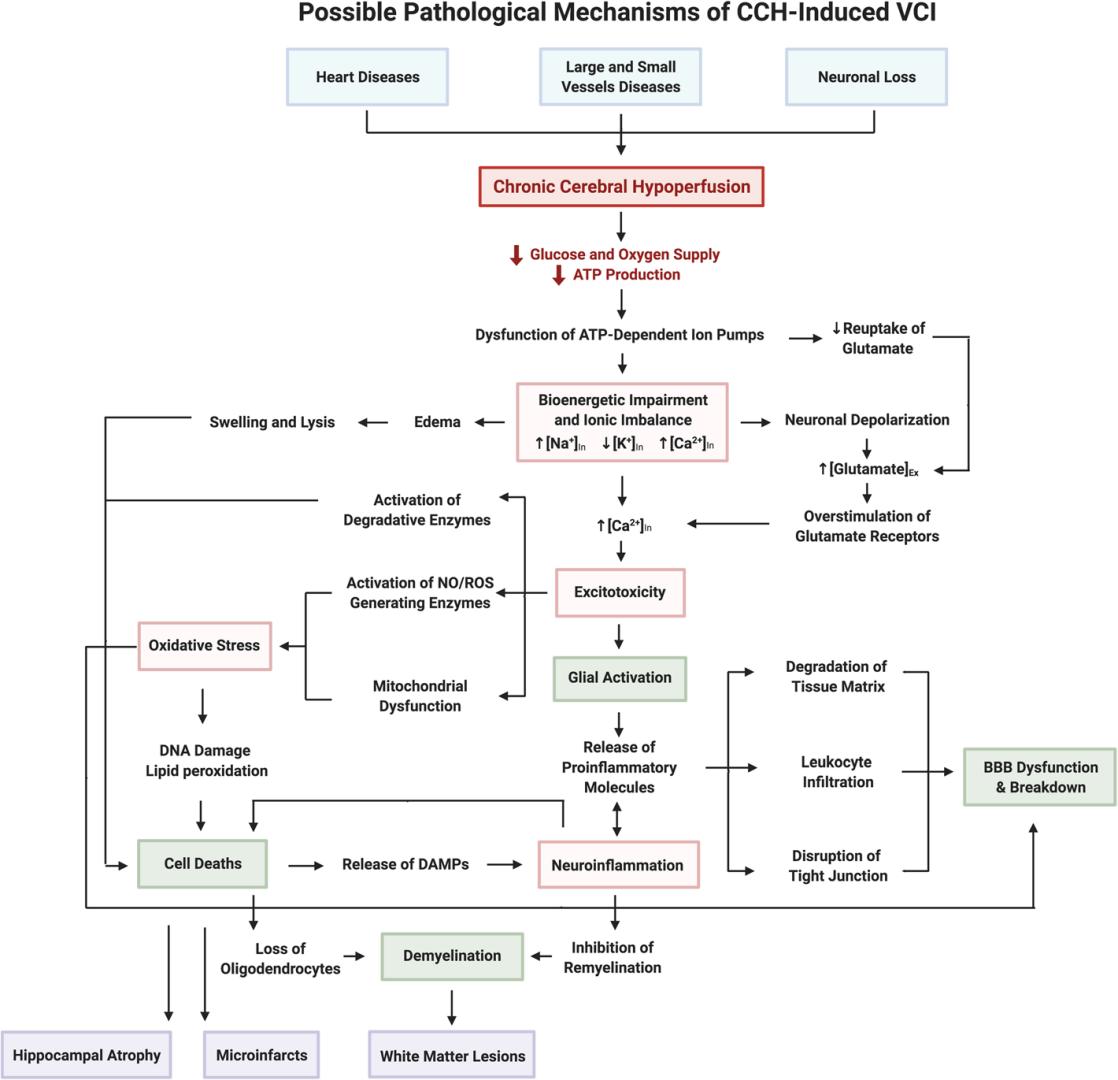
A schematic diagram illustrating the possible pathological mechanisms of VCI. Cardiovascular disease is major contributor to early cerebral blood flow reduction in the disease progression of VCI. These conditions include heart disease (i.e. coronary artery disease and arrhythmias) that impairs the ejection of blood into the blood circulation; and small and large vessel diseases (i.e. atherosclerosis and arteriosclerosis), which narrow the vascular lumen and impede blood flow. Neuronal loss results in reduced production of angiogenesis regulators, leading to neurovascular unit uncoupling. These conditions converge to cause chronic cerebral hypoperfusion that reduces delivery of glucose and oxygen to the brain leading to decreased energy (i.e. ATP) production, resulting in bioenergetic impairment. Reduced ATP levels initiate a series of pathogenic molecular and cellular mechanisms. Firstly, the function of ATP-dependent transporters (i.e. Na^+^/K^+^ ATPase) are impaired leading to ionic imbalance (i.e. Na^+^ and Ca^2+^ influx, and K^+^ efflux) across the plasma membrane resulting in anoxic depolarization within neurons causing excitotoxicity. Moreover, increased levels of intracellular Ca^2+^ activate a wide variety of calcium-dependent ROS generating pathways in the mitochondria and cytosol contributing to oxidative stress. Finally, neuroinflammation is activated as stressed or injured cells release DAMPs that bind to PRRs to induce an inflammatory response. Under CCH, these molecular mechanisms influence each other within different cell types that result in the following pathogenic cellular mechanisms: glial activation, BBB dysfunction, cell death and demyelination. As pathogenic cellular mechanisms accumulate, they synergistically drive further damage eventually causing structural damage such as white matter lesions, microinfarcts and hippocampal atrophy. Each of these structural changes cause disruption to the neuronal network and functional connectivity that eventually leads to cognitive decline. Abbreviations: VCI, vascular cognitive impairment; ATP, adenosine triphosphate; ROS, reactive oxygen species; DAMPs, damage-associated molecular patterns; CCH, chronic cerebral hypoperfusion; BBB, blood brain barrier

Disruptions to CBF directly results in reduced glucose and oxygen supply, leading to immediate bioenergetic impairment and ionic imbalance, and development of excitotoxicity, oxidative stress and inflammation (Fig. 1).

#### Bioenergetic impairment and ionic imbalance

Bioenergetic impairment refers to the disruption of cellular energy metabolism. Inadequate blood supply due to CCH is likely to result in bioenergetic impairments as neurons are unable to produce sufficient adenosine triphosphate (ATP) for normal cellular functions [35, 36]. Reduction in ATP production leads to compromised function of ATP-dependent ion channels such as the Na^+^/K^+^ and Ca^2+^ pump. The ion channels’ inability to maintain an ionic balance results in a net Na^+^ and Ca^2+^ influx, and K^+^ efflux across the plasma membrane. Consequently, this increases the resting membrane potential to threshold, leading to unregulated depolarizations known as anoxic depolarization in neurons [37, 38].

Studies have provided evidence suggesting bioenergetic impairment and ionic imbalance caused by CCH [39–41]. Mitochondrial dysfunction has been reported to be evident in VCI, whereby the expression and activity of mitochondrial enzymes that are vital for ATP production and cellular bioenergetics are reduced in a CCH rodent model of VCI [39]. Several energy metabolites including ATP were significantly reduced upon CCH [40]. Impairment in Na^+^/K^+^ homeostasis has also been observed with CCH, with reduced blood flow leading to increased intracellular Na^+^ concentration and decreased in intracellular K^+^ concentration [35]. Pharmacological inhibition of Ca^2+^ influx offers neuroprotective effects on hippocampal neurons under CCH, demonstrating the significance of Ca^2+^ influx in mediating CCH-induced injuries [42].

#### Excitotoxicity

Excitotoxicity is the damage or death of neurons from the uncontrolled stimulation of excitatory glutamate receptors and is prevalent during CCH. As neurons undergo anoxic depolarization during cerebral hypoperfusion, there is a resulting influx of Ca^2+^ ions into the presynaptic neuronal terminals, culminating in a massive release of the excitatory neurotransmitter glutamate into the synaptic cleft [35, 43]. Consequently, postsynaptic glutamate receptors on surrounding neurons become overstimulated, causing a large influx of Na^+^ and Ca^2+^ ions that trigger the propagation of an uncontrolled downstream cytotoxic cascade. Accumulation of intracellular Na^+^ creates an osmotic pressure for water to enter the neuron, leading to cytotoxic swelling and lysis [35, 44, 45]. High levels of intracellular Ca^2+^ activate downstream catabolic enzymes such as endonuclease and calpain, which degrade key cellular components such as nuclear DNA and the extracellular matrix, respectively, potentially resulting in either apoptosis and necrosis depending on CCH severity [38, 46]. Studies have shown the therapeutic potential of interventions targeting excitotoxicity, including memantine, glutamate receptor antagonist and calcium channel blockers, in ameliorating VCI for both patients and rodent models of VaD [47–50].

#### Oxidative stress

Oxidative stress occurs when the balance of reactive oxygen species (ROS) and antioxidants disrupted and is increasingly implicated in VaD. Upon disruption of CBF, the mitochondrial electron transport chain is disturbed, electron leakage occurs and reaction with oxygen produces a ROS called superoxide (O_2_^−^) [38, 51, 52]. Disruption of calcium homeostasis during CCH-induced excitotoxicity can also trigger ROS overproduction through depolarization of the mitochondrial membrane and activation of downstream ROS-generating enzymes such as NADPH (nicotinamide adenine dinucleotide phosphate) oxidases and xanthine oxidase [53, 54]. It was recently suggested that a major source of ROS production in the cytosol was activated NADPH oxidase in VCI, with enzyme inhibition capable of ameliorating cognitive impairment in a CCH rodent model [55]. Moreover, O_2_^−^ can spontaneously undergo a series of dismutation reactions to form other types of ROS such as hydrogen peroxide (H_2_O_2_) and hydroxyl radical (OH^−^) [56, 57].

Oxidative stress can cause DNA damage and induce oxidation of lipids and proteins that eventually results in apoptotic death [58]. Elevated hydrogen peroxide levels were observed in isolated mitochondria from the brain of rodent models of VCI, with an increase proportionate to the duration of CCH [39]. Several markers of oxidative stress are elevated in VCI patients, such as lipid peroxidation and oxidized DNA, coupled with reduced antioxidant levels in the plasma [59–61].

#### Neuroinflammation

Neuroinflammation is characterized by an increased production of proinflammatory cytokines and chemokines by resident brain cells such as microglia and astrocytes, together with infiltration of peripheral immune cells into the CNS [62, 63]. This is usually in response to pathogens or to a variety of pathophysiological mechanisms such as bioenergetic imbalance, excitotoxity, mitochondrial dysfunction and oxidative stress causing stress or injury to neurons, glial and vascular endothelial cells during CCH. Consequently, danger signals such as damage-associated molecular patterns (DAMPs) that are released into the extracellular environment are able to initiate local and systemic inflammation [62, 63].

The presence of chronic inflammation has been reported in VCI patients during pre-clinical, clinical and severe stages of VaD [64–67]. These studies reported elevated levels of classic inflammatory mediators such as interleukin-1 beta (IL-1β), interleukin-6 (IL-6), TNFα and C-reactive protein (CRP) that propagate inflammation, leading to degradation of the tissue matrix and infiltration of peripheral immune cells causing various forms of cell death [64–67]. Studies in animal models suggest that CCH can induce both acute and chronic neuroinflammation that damage the myelin sheath, blood-brain barrier (BBB) and grey matter via oligodendrocyte loss, endothelial cell dysfunction, and apoptotic and necrotic cell death of the neurovascular unit [68–71]. Furthermore, the complement system may also be involved in driving late stage neuroinflammatory processes via complement factor 5a (C5a), as elimination of C5a in a VCI mouse model exerts a protective effect against CCH-induced injury [72].

### Pathogenic cellular mechanisms of CCH

The aforementioned molecular mechanisms initiated by CCH are critical drivers of subsequent pathogenic cellular mechanisms in VCI such as glial activation, blood-brain barrier dysfunction, cell death and demyelination (Fig. 1).

#### Glial activation

Glial activation occurs when resident immune cells switch from a resting state to an activated state to initiate a series of changes in glial function following cellular stress and injury in the brain. In general, microglia and astrocytes are considered the main resident immune-associated cell types in the brain that respond readily to microenvironmental disturbances. CCH can trigger glial activation through ionic imbalance, oxidative stress and neuroinflammation [23, 73, 74] .

Several studies of brains from VCI have shown the presence of reactive astrocytes and microglia in the areas surrounding lesions, alongside markers of oxidative stress and inflammation [75–77]. These activated glial cells are likely to be involved in the pathophysiology of VCI via several mechanisms. Firstly, they initiate and facilitate neuroinflammation, leading to cellular injury and leukocyte infiltration into the brain [78, 79]. Secondly, inflammation suppresses the pro-survival action of endothelial cells on neurons by reducing neurotrophic signaling leading to endothelial cell atrophy and microvascular rarefaction [80, 81]. Thirdly, activated microglia release proinflammatory cytokines and chemokines that disrupt BBB integrity by redistributing tight junction proteins and reorganizing the actin cytoskeleton in microvascular endothelial cells in the brain [82, 83]. Finally, glial activation contributes to demyelination by impeding remyelination, as reactive astrocytes and microglia surrounding the white matter lesion release IL-1β and hyaluronan to inhibit maturation of oligodendrocytes during CCH [69, 84].

#### Blood-Brain barrier dysfunction and breakdown

Blood-brain barrier (BBB) dysfunction is when the integrity of the highly selective semipermeable border surrounding the brain parenchyma is compromised. BBB function involves coordinated interactions between endothelial cells and pericytes regulating the selective diffusion of substances into the brain parenchyma. In particular, it is regulated by tight junction proteins (TJPs) located between endothelial cells, with the support of pericytes attached [85, 86]. Disruption of TJP arrangement or distribution loosens the interaction between adjacent endothelial cells, compromising the physical barrier and increasing its permeability to foreign substances [87, 88]. CCH-induced oxidative stress and inflammation contribute significantly to BBB dysfunction by reducing the density of TJPs on the endothelial membrane [89–91]. Inflammation can also upregulate cell adhesion molecules (i.e. Intercellular Adhesion Molecule (ICAM) and Vascular Cell Adhesion Molecule (VCAM) to facilitate the infiltration of peripheral immune cells across the BBB to release additional ROS and proinflammatory cytokines [90, 92, 93]. Finally, proinflammatory cytokines can upregulate gene expression of matrix metalloproteinases, MMP2 and MMP9, that can also degrade the extracellular matrix contributing to BBB dysfunction [94, 95].

Dysfunction of the BBB is increasingly implicated in VCI [96, 97]. In multiple mouse models of CCH, impairment of the BBB is observed through increased vascular permeability of intravascular Evans blue dye and horseradish peroxidase (HRP) into the brain parenchyma [91, 98, 99]. Several studies have also shown that CCH reduced pericyte coverage on capillaries, contributing to BBB dysfunction via endothelial transcytosis [91, 100, 101]. Moreover, the cerebrospinal fluid/plasma albumin ratio, another indicator of BBB damage, has also been reported to be elevated in VCI patients relative to healthy controls, with the severity of BBB damage corresponding to white matter lesions [102]. While the nature and extent of BBB disruption in the pathogenesis of VCI remains to be fully elucidated, it is currently thought to be mediated by oxidative stress and neuroinflammation [103, 104].

#### Cell death: necrosis and apoptosis

The aforementioned pathogenic molecular mechanisms caused by CCH, including bioenergetic imbalance, excitotoxicity, oxidative stress and inflammation, can disrupt the integrity of the neurovascular unit leading to programmed neuronal and glial death [38, 58, 105]. In particular, accumulation of Ca^2+^ in the cytosol from excitotoxicity can activate catabolic enzymes that cleave DNA and hydrolyze cellular cytoskeletal proteins to cause apoptosis [46, 106, 107]. Similarly, CCH-induced oxidative stress and neuroinflammation can also activate cell death pathways. For example, H_2_O_2_ can trigger necrosis and apoptosis via the modulation of activator protein-1 (AP-1) and B-cell lymphoma-2 (Bcl-2) family proteins, respectively [108, 109]. The proinflammatory cytokine, TNF-α, is a critical ligand for death receptors that can activate pro-apoptotic caspase-8 and -3 in the extrinsic apoptotic pathway during CCH [110].

Necrosis and apoptosis are two types of cell death that have been established to occur clinically in VCI. In VCI, necrotic cell death can be observed within lacunar infarcts formed when the brain tissue is exposed to total or partial reduction of blood flow [111, 112]. Apoptosis is a highly conserved cell death pathway involving the family of cysteine-dependent aspartate specific proteases (Caspases), which induces DNA fragmentation and phagocytic signaling [113, 114]. Apoptosis has also been observed in cerebral autosomal dominant arteriopathy with CADASIL patients and various mouse models of VCI [115–117]. In addition to necrosis and apoptosis, recent studies have demonstrated inflammasome mediated necroptotic and pyroptotic forms of cell death during CCH, suggesting its potential involvement in VCI [66, 71, 118, 119].

#### Demyelination

Demyelination refers to a condition where the protective myelin sheath that surrounds neuronal axons is damaged and degraded. It is commonly observed within the deep white matter in patients with small vessel disease, leading to cognitive decline in the aged brain [120, 121].

CCH can result in demyelination induced by a hypoxic-ischemic environment and inflammation [121, 122]. Hypoxic conditions trigger the activation of the hypoxia-inducible factor-1 (HIF-1) regulatory pathway leading to inflammation and apoptotic cell death [76, 123]. Inflammation activates glial cells to release inflammatory mediators such as TNF-α, matrix metalloproteinases (MMPs) and serine proteases that damage the myelin sheath [77, 124, 125]. Active proteases (i.e. MMP-1, MMP-2, MMP-3, MMP-7, MMP-9 and Calpain) induced by CBF disruption degrade myelin basic protein (MPB), and disrupt the polymeric network within the myelin sheath [95, 124, 126]. Moreover, CCH-induced inflammation can initiate activation of apoptosis and pyroptosis in oligodendrocytes, that may attenuate myelin synthesis and repair, and exacerbate demyelination [71].

### Pathological Structural features and cognitive impairment from CCH

Several structural pathological features are observed in the brain during VCI. Advances in neuroimaging have allowed detection of white matter lesions, lacunes, microinfarcts, microbleeds and enlarged periventricular spaces, which are now considered standard diagnostic features of VCI [15, 127, 128]. In this section, several key pathogenic structural damages and cognitive impairment associated with CCH will be discussed (Fig. 1).

#### White matter Lesions

White matter lesions (WMLs) are regions in the brain parenchyma with demyelination in the white matter that appear as hyperintensities (without cavitation) on T2-weighted MRI images [128]. WMLs are primarily formed from axonal demyelination that is usually produced from the loss of oligodendrocytes [129, 130], and accompanied by both glial activation [131, 132] and loss of axon-glial integrity [133]. These features are a reflection of the underlying mechanisms of CCH-induced WML formation. As discussed above, CCH triggers molecular mechanisms that contribute to glial activation, cell death and demyelination. As cerebral hypoperfusion persists, these injuries accumulate at the cellular level, especially in oligodendrocytes within the white matter region and surrounding glial cells.

WMLs are a significant contributor to cognitive decline and are a prominent feature of VCI [134–136]. WMLs disrupt the functional connections between the cortical and subcortical regions, affecting cognitive function and emotions [137, 138]. WMLs also reflect loss of cholinergic neurons, compromising the neurotransmitter system, eventually leading to cognitive decline in VCI patients [134, 139]. The main mechanisms underlying the formation of WMLs in humans are critical stenosis and hypoperfusion of medullary arterioles in small vessel disease (i.e. arteriolosclerosis) and hypotensive episodes [140–142]. White matter regions with lower CBF developed into WMLs in a longitudinal study [143]. The positive association between CCH and a decline in cognitive function is particularly profound in patients with severe WMLs [28]. In mouse models, CCH resulted in similar white matter rarefaction and lesion formation together with cognitive impairment, further emphasizing the common role of WMLs in VCI [71].

#### Microinfarcts

Microinfarcts are small fluid-filled spaces/lesions that appear in large numbers within the cortical and subcortical brain regions. The formation of microinfarcts is usually caused by the activation of necrotic and programmed cell death pathways in neurons and glial cells, and commonly associated with neuronal loss, gliosis and axonal damage [144–146]. This process is often accompanied by the migration of glial and peripheral immune cells, such as macrophages, to the necrotic site of injury. Immune cells phagocytose damaged cells, and astrocytes proliferate and undergo gliosis to form a barrier around the lesion to limit the spread of necrosis [145–147].

Microinfarcts are more prominent in VCI than in other demented patients [148]. In VCI, microinfarcts are associated with cerebral amyloid angiopathy and reduced cerebral perfusion [22, 120]. Several post-mortem studies have demonstrated that hypoperfusion initiates and promotes the progression of microinfarct formation in brain areas vulnerable to hypoperfusion (i.e. watershed cortical region) [149, 150]. Microinfarcts affect the brain structural network, leading to impaired performance in various cognitive domains [151, 152]. Microinfarcts also cause primary disruption to local tissue function, secondary inflammation and axonal disorganization to the white matter tracts, further exacerbating damage to brain circuits and function [153–155].

#### Hippocampal atrophy

Hippocampal atrophy refers to the loss of neurons and neuronal volume in the hippocampus. It is a classic marker for AD, but can also be observed in VCI patients. The degree of atrophy has been estimated to be 16.6% in AD and 11.6% in VCI [156–158]. The hippocampus is a complex brain region that is highly sensitive and vulnerable to insults caused by reduced cerebral perfusion or hypoxia, which stimulate a number of pathogenic mechanisms such as excitotoxicity, oxidative stress, and inflammation to activate cell death pathways [159, 160], leading to hippocampal atrophy. Animal studies have shown that with CCH, the hippocampus displays acute neuronal damage and cell death originating from the CA4 area, to CA2 and CA3, with the CA1 area being the last zone affected [71, 161, 162]. Decline in memory performance, spatial navigation and visuospatial functions have been observed in patients with hippocampal atrophy [163, 164]. While the direct mechanistic explanation for these associations in VCI has not been established, it is suggested that reduced neuronal capacity in the hippocampal area lowers its connectivity with other brain regions [163–165]. Decreased levels of the synaptic protein synaptophysin has been reported in VCI patients compared to healthy subjects, suggesting a potential deficit in synaptic transmission upon hippocampal atrophy [144, 166] .

### Cognitive dysfunction in VCI

VCI encompasses a broad spectrum of cognitive dysfunctions ranging from subjective cognitive impairment, mild cognitive impairment to dementia (VaD) [8, 13]. The underlying pathophysiological mechanisms responsible for CCH-induced cognitive impairment may be extensive and severe. CCH initiates several cellular mechanisms - glial activation, BBB dysfunction, cell death and demyelination, via the activation of numerous pathogenic molecular mechanisms, including bioenergetic impairment and ionic imbalance, excitotoxicity, oxidative stress and neuroinflammation in different types of brain cells. These mechanisms all contribute to structural damage such as WMLs, microinfarcts and hippocampal atrophy. The influence of these structural damages on cognitive function was discussed previously.

Several guidelines have proposed that VCI is a syndrome with evidence of either clinical stroke or subclinical vascular brain injury, and cognitive impairment affecting at least one cognitive domain [8, 15, 167]. The cognitive domains involved in diagnosing VCI are executive/attention, memory, language, and visuospatial function [8]. Executive dysfunction is a well characterized neurological feature of VaD and can be present in VCI patients who are not demented [168, 169]. Impairments to executive functions are heavily associated with lesions and damage within the frontal lobes or downstream frontal-subcortical circuits of VCI/VaD patients [170, 171]. Memory impairment is a key feature of AD and is also important in VaD. Studies have shown that VaD patients experienced greater impairment in semantic memory than AD patients, possibly due to the inability to retrieve information from short and long-term memory [172–174]. Impairments in language and visuospatial functions can also be observed in VCI. When presented with a picture description task, VaD patients exhibit lower semantic content production while maintaining a fluency comparable to healthy patients [175]. VaD patients show impairments in most of the tests associated with visuospatial tasks, indicating a deficit in constructional and visuoperceptual ability [174, 176].

## Inflammation – a critical molecular mechanism and a bridge between CCH and various cellular mechanisms

Several key events have been identified in the pathophysiology of VCI, as briefly summarized above. However, whether these events are causative or merely consequential to the progression of VCI has yet to be conclusively validated. A comprehensive understanding on the pathogenesis of VCI will not only pave the way for development of interventions, but allow us to target VCI with maximum efficacy [8]. In addition, interventions targeting an early temporal event in the pathogenesis of VCI would better attenuate other repercussions elicited by the many late temporal events in the pathogenesis of VCI. Hence, there is an increasing focus on exploring the temporal profile of events in the pathogenesis of VCI. The current notion regarding VCI remains that the underlying root cause is the initial disruption to CBF, with a cascade of events leading to cognitive impairments [20–22, 26, 177]. Specifically, several lines of evidence have highlighted a plausible causative role of neuroinflammation as an early temporal event that then influences other mechanisms in contributing to the pathogenesis of VCI [64–67, 77].

Neuroinflammation has been implicated in dementia, and is associated with a decline in cognitive functions and functional connectivity in demented patients [178, 179]. Elevated levels of inflammatory markers, including the highly sensitive CRP and IL-6, IL-8 and IL-1β, were found in brain tissues and peripheral samples of demented patients [65, 66, 180–182]. Some of them (e.g. IL-6 and CRP) were even associated with an increased risk of dementia, including VaD [181, 182]. However, a few studies demonstrated lower levels of inflammatory mediators in patients with dementia. For example, Chen et al. and Mulugeta et al. showed that normal controls had significantly higher levels of IL-6 and IL-8 in different parts of the brain when compared to AD, mixed dementia or VaD patients [183, 184]. It was also observed that IL-6 and IL-8 levels were lower in the cerebrospinal fluid and plasma of patients with AD [185, 186]. While the results are controversial, it is essential to consider the following points. Firstly, the profile of cytokines may depend on the stage and progression of the disease [187, 188]. Secondly, some of these inflammatory cytokines, such as IL-6 and TNF-α, may exhibit pleiotropic actions with known pro- or anti-inflammatory effects [187, 188], which adds more complexity to the interpretation of these studies. Lastly, heterogeneity of post-mortem samples and difficult application of the detection methods for proinflammatory mediators in the brain and peripheral samples may account for the contradictory conclusions of some studies [189–191]. Despite these variable results, inflammation is still considered one of the critical underlying mechanisms of dementia. More importantly, the levels of these proinflammatory mediators were shown to be elevated even before the clinical onset of VaD [67] suggesting that neuroinflammation may be involved in both early and late stages of VCI disease progression.

The associations between neuroinflammation and impairments in cognitive function may stem from its involvement in several key events in the pathogenesis of VCI, such as glial activation, BBB dysfunction, cell death, demyelination and WML formation. The influence of neuroinflammation on these events through the action of the inflammasome signaling pathway will be discussed in the following section.

##  Inflammasome signaling pathway: linking IL-1 to VCI

Among the numerous inflammatory mediators implicated in VCI, one group of proinflammatory cytokines is prominent: the interleukin-1 (IL-1) family. Amongst the IL-1 family members, both IL-1β and IL-18 are increasingly implicated in the progression of VCI. Elevated levels of both IL-1β and IL-18 have been reported in the serum of VaD patients [64, 65, 192]. While there could be an involvement of systemic inflammation in the studies of serum samples, post-mortem studies of different brain tissues showed that the level of IL-1β was higher in the frontal cortex and hippocampus of VaD patients in comparison to controls [66, 193] indicating that IL-1β may be strongly involved in the process of neuroinflammation during VCI. The production and maturation of both IL-1β and IL-18 is primarily driven from a major arm of the innate immune system termed the inflammasome signaling pathway [194]. As such, this pathway is likely to serve as a critical point in regulating production of IL-1 family cytokines during the pathogenesis of VCI.

### Overview of the Inflammasome signaling pathway

Inflammasomes are macromolecular protein complexes that are essential signaling platforms capable of detecting pathogenic signals via pathogen-associated molecular patterns (PAMPs) and endogenous sterile stressors via damage-associated molecular patterns (DAMPs) [38]. Upon detection of PAMPs or DAMPs, the inflammasome complex is activated to trigger downstream inflammatory cascades such as the production of inflammatory cytokines. Given its sterile nature, neuroinflammation in VCI can be considered exclusively driven by DAMPs. The two main characterized inflammasome signaling pathways are the canonical and non-canonical pathways, each of which involves two steps to achieve inflammasome signaling: priming and activation [38, 195, 196].

**Fig. 2:**
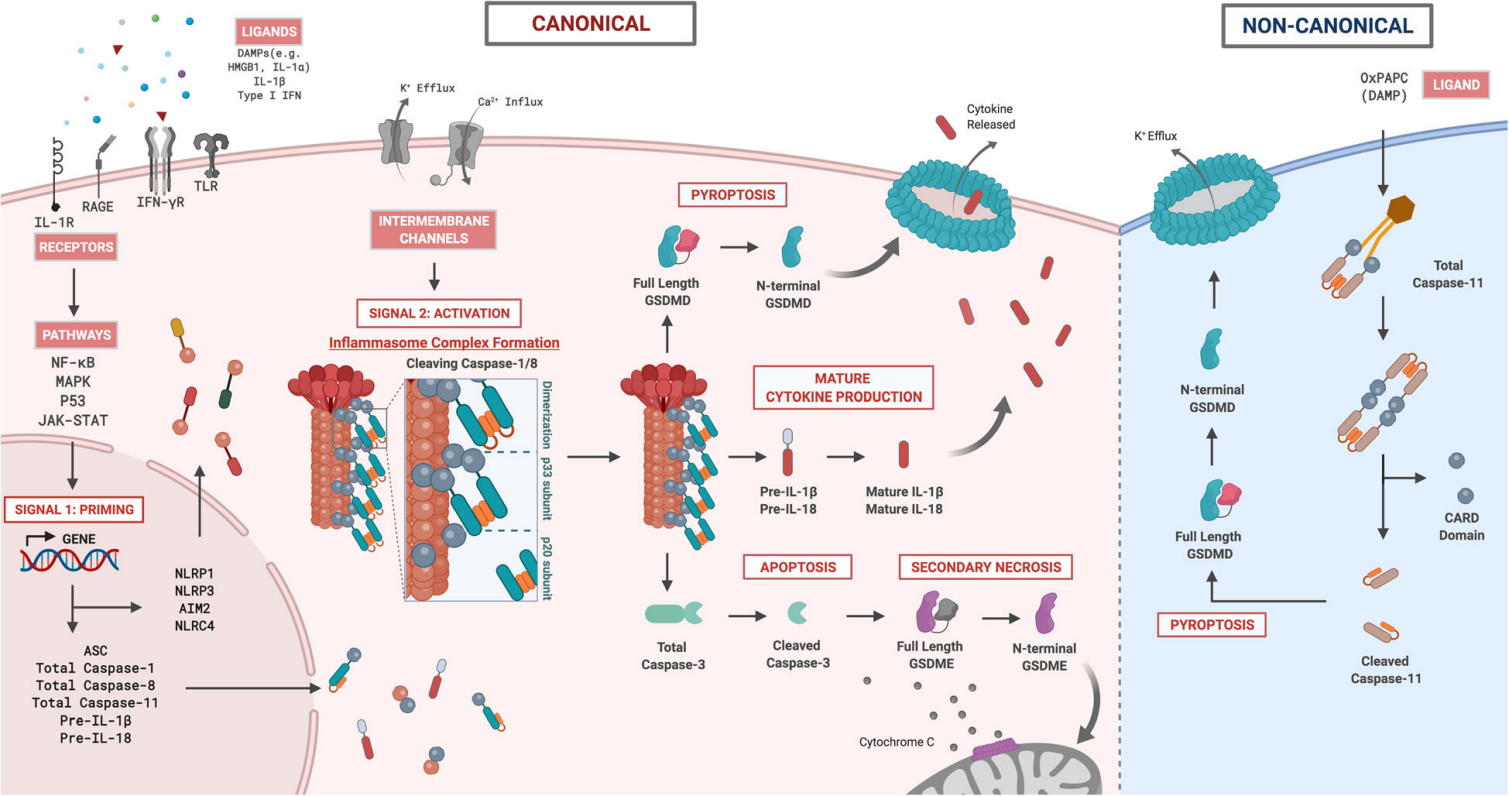
A schematic diagram illustrating the canonical and non-canonical inflammasome signaling pathways in the brain during chronic cerebral hypoperfusion. In the canonical inflammasome pathway, two signals – priming and activation are involved. The first signal is the priming step whereby endogenous extracellular ligands (DAMPs) are able to bind onto its respective pattern recognition receptors (i.e. TLR, RAGE, IFN-γR, IL-1R) on neighbouring cells, activating several downstream regulatory pathways (i.e. NF-κB, MAPK, P53 and JAK-STAT), leading to increased gene expression of inflammasome components (i.e. receptors, adaptor and effector proteins) and both precursor IL-1β and IL-18 in the cytosol. Following priming, a second signal is required to activate the inflammasome receptor(s) to form a macromolecular platform that recruits the adaptor protein (i.e. ASC) and effector proteins (i.e. total caspase-1 and -8) to form a multi-protein complex termed an inflammasome. In the inflammasome complex, total caspase-1 and -8 undergo proximity-induced activation to form active cleaved caspase-1 and -8 that initiates several catalytic functions. First, cleaved caspase-1 and -8 induces mature cytokine production by cleaving precursors IL-1β and IL-18 into active mature IL-1β and IL-18 proinflammatory cytokines. Second, cleaved caspase-1 and -8 are able to initiate an inflammatory form of cell death by cleaving GSDMD-FL into GSDMD-NT. As more GSDMD-NTs are produced in the cytosol, these fragments self-oligomerize onto the plasma membrane to form a pore to facilitate the influx of water molecules to induce a lytic form of cell death known as pyroptosis. Third, cleaved caspase-1 and -8 can trigger apoptosis by cleaving total caspase-3 into active cleaved caspase-3. Moreover, active cleaved caspase-3 can also initiate another form of cell death known as secondary necrosis by cleaving GSDME-FL into GSDME-NT. Similar to GSDMD-NT, GSDME-NT can self-oligomerize to form pores on the plasma membrane; in addition to forming pores on the mitochondrial membrane, which results in cytochrome c release, further exacerbating apoptosis. In the non-canonical inflammasome pathway, total caspase-11 can be activated by binding to an endogenous ligand (i.e. OxPAPC) that allows oligomerization of total caspase-11. Such oligomerization initiates the proximity-induced activation of total caspase-11 to form active cleaved caspase-11. The non-canonical effector protein, cleaved caspase-11, can also directly cleave GSDMD-FL into GSDMD-NT to cause pore formation. It has been shown that K^+^ efflux resulting from pore formation can serve as an activation signal for canonical NLRP3 receptor activation, indicating cross-talk between the canonical and non-canonical inflammasome signalling pathways. Abbreviations: DAMPs, damage associated molecular patterns; HMGB1, high mobility group box protein 1; IL, interleukin; IFN, interferon; TLR, toll-like receptor; RAGE, receptor for advanced glycation end products; NF-κB, nuclear factor kappa-light-chain enhancer of activated B cells; MAPK, mitogen activated protein kinase; JAK/STAT, janus kinase-signal transducer and activator of transcription; Pre, precursor; GSDMD, gasdermin D; GSDME, gasdermin E

The canonical inflammasome signaling pathway typically leads to the activation of caspase-1 and -8 (Fig. 2). The priming step (i.e. Signal 1) is initiated by the presence of DAMPs (e.g. HMGB1, IL-1α), which can activate various extracellular PRRs including toll-like receptors (TLR; TLR2, TLR4), receptor for advanced glycation end-products (RAGE) and interleukin-1 receptor 1 (IL-1R1) [195, 197], leading to downstream activation of NF-κB, MAPK, p53 and JAK-STAT pathways [38, 195, 198]. The main purpose of priming is to increase gene expression of key inflammasome components (i.e. receptor/sensor, adaptor and effector) and precursors of IL-1β and IL-18 in the cytosol [199, 200]. Priming can be independent of transcriptional expression whereby post-translational modifications (i.e. phosphorylation and de-ubiquitination) are essential to “license” inflammasome activation [201]. The activation step (i.e. Signal 2) involves stimulation of cytosolic inflammasome receptors/sensors that can be triggered by various DAMPs and/or disturbances in the cellular microenvironment, resulting in assembly and activation of the canonical inflammasome complex to facilitate activation of effectors caspase-1 and -8 [194, 202]. The NLRP3 complex is arguably the best characterized inflammasome, and along with the NLRP1, NLRC4 and AIM2 inflammasome complexes facilitates the self-cleavage of total caspase-1 and -8 into biologically active cleaved caspase-1 and -8, respectively [194, 195, 203, 204]. Both cleaved caspase-1 and -8 can cleave precursors of both IL-1β and IL-18 into mature proinflammatory cytokines that can then amplify downstream inflammation [205, 206]. Cleaved caspase-1 and -8 are also implicated in the activation of programmed cell death pathways such apoptosis and pyroptosis [207–209], and will be discussed below.

The non-canonical inflammasome signaling pathway leads to the activation of the mouse homolog caspase-11, with the corresponding human homologs being caspase-4 and -5 (Fig. 2). It shares some similarities to the canonical activation of caspase-1, although the non-canonical pathway possesses some unique characteristics. Transcriptional priming is also present for caspase-11 in the non-canonical inflammasome signaling pathway, mediated by extracellular PRRs, including Toll-Like Receptors (TLRs) (i.e. TLR2, TLR4), RAGE and IL-1R1 [195, 197]. Total caspase-11 can detect cytosolic lipopolysaccharides (LPS) from gram-negative bacteria, and oligomerize to form a macromolecular complex of full length caspase-11 components. Subsequently, full length caspase-11 undergoes autoproteolytic cleavage via proximity-induced activation and conversion to its active cleaved form. Cleaved caspase-11 can induce pyroptosis and also indirect maturation of IL-1β and IL-18, in a caspase-1-dependent manner via induction of K^+^ efflux [210, 211]. Low intracellular K^+^ levels can activate NLRP1 and NLRP3 inflammasomes, thereby linking the non-canonical pathway to the canonical inflammasome pathway [212, 213]. Caspase-11 can also be activated by a class of DAMPs known as oxidized 1-palmitoyl-2-arachidonyl-*sn*-glycero-3-phosphorylcholine (oxPAPC), although oxPAPC upregulates the production and secretion of IL-1β, but does not induce pyroptosis [214].

### Molecular structure of Inflammasome components

**Fig. 3:**
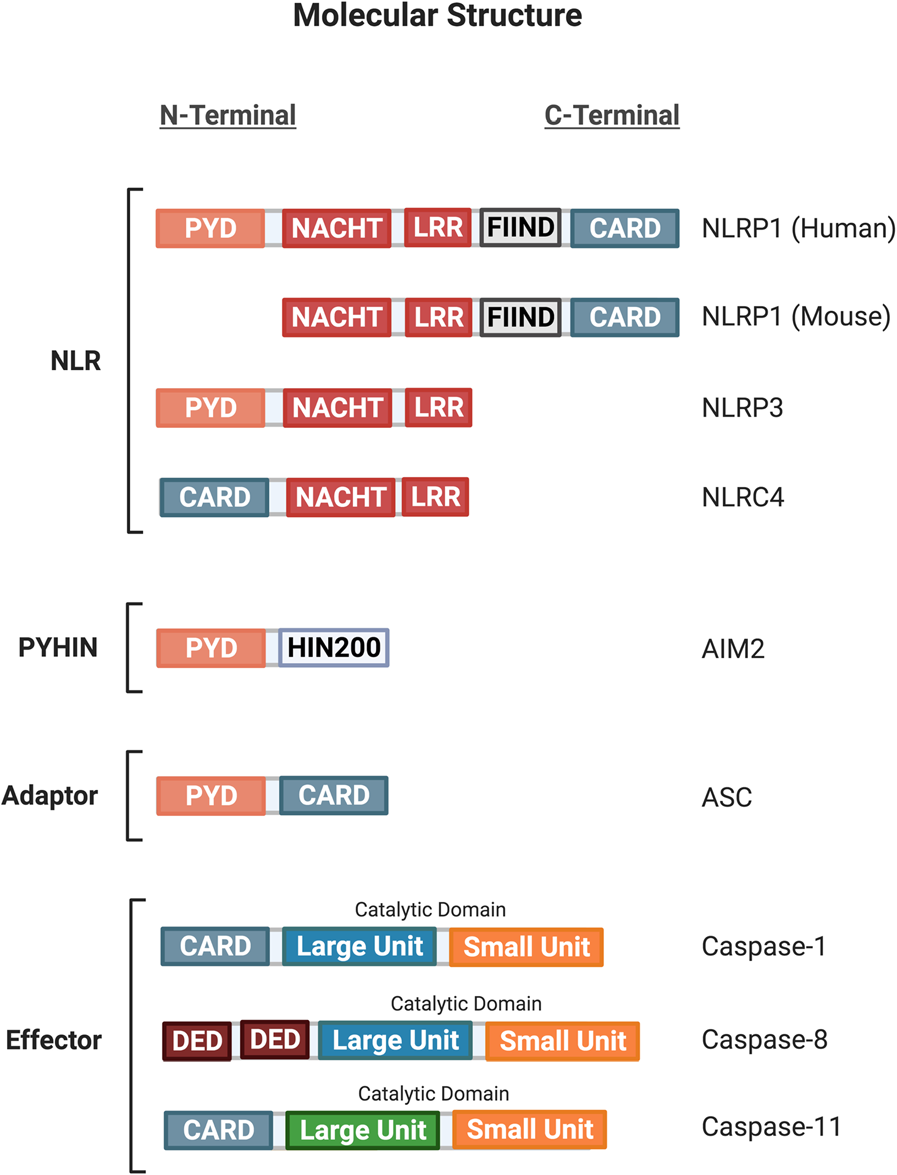
A schematic diagram illustrating the molecular structure of inflammasome receptor, adaptor and effector components. Members of the NLR family share similar structures: the NACHT domain is responsible for NLR oligomerization, while LRR is the inhibitory unit of the NACHT domain, keeping the receptor in its inactive state. The two other critical domains are the PYD domain and the caspase activation and recruitment domain (CARD) to facilitate interactions with other inflammasome components with similar domains to form the NLRP1, NLRP3 and NLRC4 inflammasome complex. Under the PYHIN family, the AIM2 inflammasome receptor has a PYD domain for adaptor binding and a HIN200 domain for dsDNA ligand binding. The adaptor protein ASC has both the PYD and CARD domain, serving as a linker protein between the inflammasome receptor and effector protein components. The three effector protein components share similar catalytic units (i.e. large and small units) and an N-terminal domain (i.e. CARD or DED) for inflammasome complex binding. Abbreviations: NLR, nucleotide-binding oligomerization domain-like receptor; NACHT, NAIP (neuronal apoptosis inhibitor protein) C2TA (class 2 transcription activator, of the MHC) HET-E (heterokaryon incompatibility) and TP1 (telomerase-associated protein 1); LRR, leucine-rich repeat; PYD, pyrin domain; CARD, caspase recruitment domain; NLRP1, NLR family pyrin domain containing 1; NLRP3, NLR family pyrin domain containing 3; NLRC4, NLR family CARD domain-containing protein 4; AIM2, absent in melanoma 2; HIN200, hematopoietic interferon-inducible nuclear proteins with a 200-amino-acid repeat; dsDNA, double-stranded DNA; ASC, apoptosis-associated speck-like protein containing a CARD; DED, death effector domain

A canonical inflammasome complex is typically comprised of three distinct components: a receptor/sensor, an adapter and effector (Fig. 3). The sensor is usually a cytosolic PRR that detects perturbations in the intracellular microenvironment that serve as the activation signal to initiate inflammasome assembly. The inflammasome complexes are named after their respective intracellular PRR, which are from two major families: the nucleotide-binding oligomerization domain-like receptor (NLR) family and the pyrin and hematopoietic expression, interferon-inducible, nuclear localization (HIN) domain-containing (PYHIN) family [38, 194]. Members of the NLR family share similar structural domains such as the NLR apoptosis inhibitory protein (NAIP), MHC class II transcription activator (CIITA), incompatibility locus protein from Podospora anserina (HET-E), and telomerase-associated protein (TP1), collectively known as NACHT, and a leucine-rich repeat (LRR) [215, 216]. The NACHT domain is responsible for NLR oligomerization, while LRR is the inhibitory unit folded onto the NACHT domain, keeping the receptor in its inactive state. The two other critical domains are the Pyrin (PYD) domain and the caspase activation and recruitment domain (CARD), which interact with other components with similar domains to form the inflammasome complex [38, 194, 217, 218]. Molecular structures of the AIM2, NLRP1, NLRP3, and NAIP-NLRC4 receptor complexes are discussed below (Fig. 3).

The NLR-pyrin domain containing 1 (NLRP1) belongs to the NLR family of PRRs, with only a single variant of NLRP1 found in humans [194, 219]. The NLRP1 receptor consists of an N-terminal PYD, NACHT, LRR, function-to-find domain (FIIND) and a C-terminal CARD domain. The FIIND domain is unique to NLRP1 in the NLR family, and may play an autoinhibitory role [219, 220] (Fig. 3). The NLR-pyrin domain containing 3 (NLRP3) belongs to the NLR family of PRRs and is perhaps the most studied of all inflammasomes. The NLRP3 receptor consists of an N-terminal PYD, NACHT and LRR domain [194, 221] (Fig. 3). The NLR apoptosis inhibitory protein (NAIP) and the NLR-CARD containing 4 (NLRC4) belong to the NLR family as they both contain NACHT and LRR domains. NAIP is distinct from other NLR proteins as they contain three baculovirus inhibitor-of-apoptosis repeats (BIR) at the N-terminal domain. Unlike the previously mentioned PRRs of the NLR family, NLRC4 contains an N-terminal CARD rather than a PYD domain, in addition to the NACHT and LRR domains [194, 222] (Fig. 3). The absent in melanoma 2 (AIM2) belongs to the PYHIN family of PRRs, and together with four other members forms the AIM2-like receptor (ALR) family of inflammasomes. AIM2 is a bipartite protein, consisting of an N-terminal PYD and a 200-amino-acid HIN domain with two oligonucleotide/ oligosaccharide-binding folds [218, 223]. The HIN domain can bind to double-stranded DNA (dsDNA) independent of its sequence, and during the absence of ligand binding can interact in an intramolecular manner with the PYD to result in autoinhibition [218] (Fig. 3).

The adapter, known as apoptosis-associated speck-like protein containing a CARD (ASC), is a protein responsible for the interactions between various inflammasome components, such as a scaffold protein that connects the PRR to the effector components. ASC contains both an N-terminal PYD domain and a C-terminal CARD domain [217, 224] (Fig. 3).

The effector protein components are a group of inflammatory caspases that catalyze a broad spectrum of substrates upon activation. Caspase-1, − 8 and − 11 play a significant role as the effectors in the inflammasome signaling pathway. Both caspase-1 and -11 contain an N-terminal CARD domain and C-terminal catalytic domain (composed of large p20 and small p10 subunits). Caspase-8 contains two death effector domains (DED) at its N-terminal and the catalytic domain at the C-terminal [225, 226]. Although these caspases are similar in structure, they differ in their domain linkers and residues in their catalytic pocket, contributing to their differential role in the inflammasome signaling pathway [227–229] (Fig. 3).

### Possible Inflammasome-inducing stimuli in VCI

**Fig. 4:**
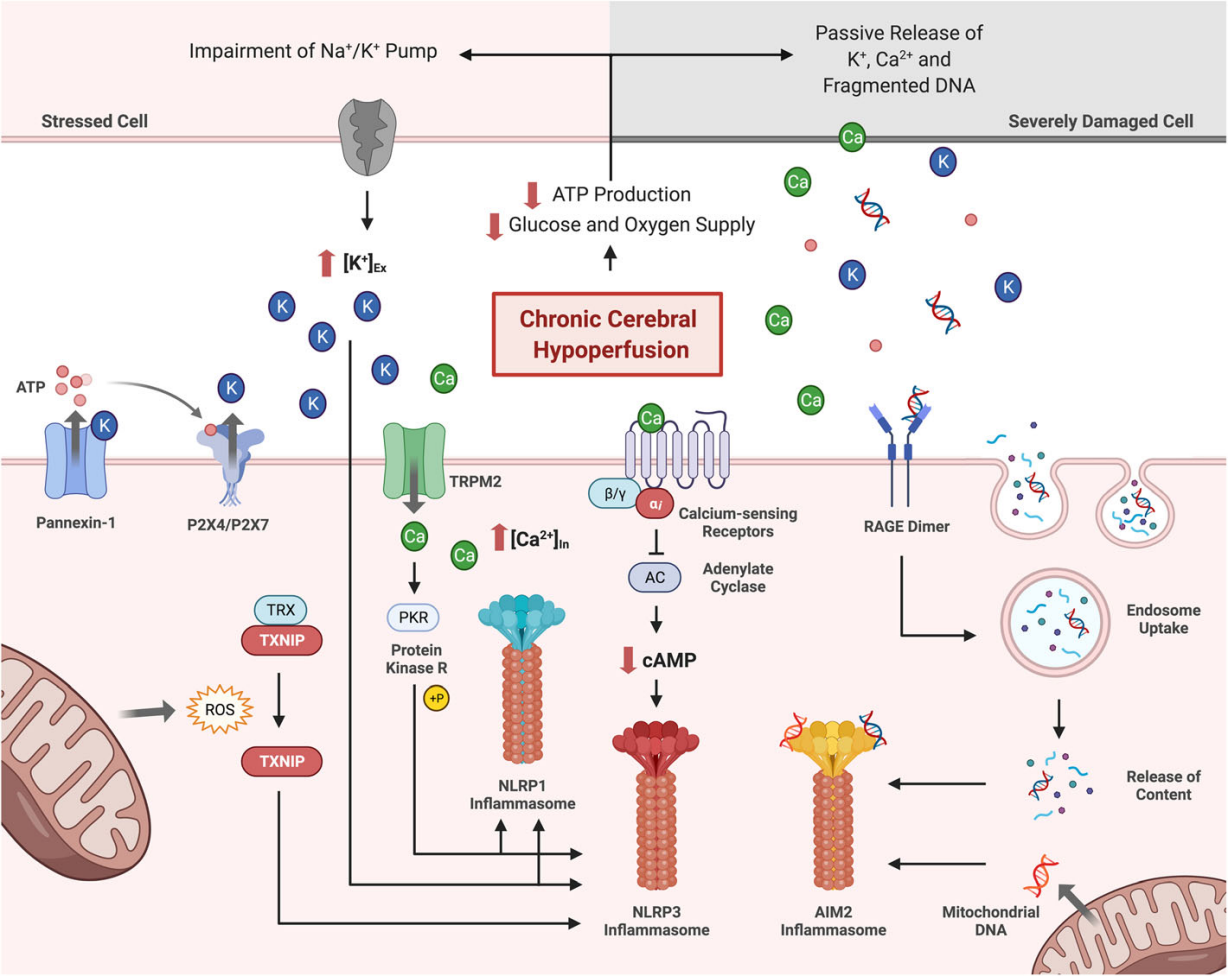
A schematic diagram illustrating potential stimuli involved in inflammasome receptor activation during CCH. The precise molecular and cellular mechanisms of inflammasome receptor activation during CCH are unknown. However, relevant studies suggest several plausible mechanisms including - decreased intracellular K^+^ concentration, increased intracellular Ca^2+^ concentration, ROS production, DNA fragmentation and oxidized mitochondrial DNA. During CCH, lower cerebral blood flow reduces ATP production, and impairs ATP-dependent transporters such as the Na^+^/K^+^-ATPase pump, leading to K^+^ accumulation in the extracellular space. Alternatively, ATP released by damaged cells can bind to the P2X4 and P2X7 receptors on neighbouring cells, leading to the receptor opening and efflux of K^+^. In addition, damaged cells can also passively release K^+^ into the extracellular environment. Extracellular K^+^ can activate Pannexin-1 channels on the plasma membrane through a mechanism independent of the membrane potential. This further promotes the release of ATP into the extracellular space, creating a positive feedback loop for K^+^ efflux. Consequently, the accumulation of extracellular K^+^ and decrease in intracellular K^+^ levels can activate the NLRP3 receptor by inducing a conformational change that promotes oligomerization. During CCH, severely damaged necrotic cells may also release Ca^2+^ into the extracellular space, activating calcium-sensing receptors (CaSRs) on neighbouring cells. Activated CaSRs inhibit the activity of adenylate cyclase, reducing the conversion of ATP to cAMP. As cAMP is an inhibitor for NLRP3, a reduction in cAMP levels in the cytosol can promote NLRP3 inflammasome activity. Ca^2+^ can also promote inflammasome activation through the TRPM2 Ca^2+^ channel during CCH. As Ca^2+^ enters the cell via the TRPM2 channel, it enables protein kinase R (PKR) in the cytoplasm to phosphorylate NLRP1 and NLRP3 receptors resulting in inflammasome activation. CCH also caused a substantial degree of oxidative stress and the production of ROS in the cell. ROS can interact with the TXNIP-TRX complex to release TXNIP from TRX, allowing it to bind to the NLRP3 receptor for subsequent inflammasome activation. CCH induces AIM2 inflammasome activation via the production and release of fragmented dsDNA. Severely damaged cells and mitochondria are the source of fragmented dsDNA during CCH. While intracellular mitochondrial dsDNA interacts directly with the AIM2 receptor in the cytosol, extracellular dsDNA enters the cell via the facilitation of RAGE. When RAGE detects the presence of dsDNA in extracellular space, it promotes endosomal DNA uptake of the cell. The dsDNA will then bind onto the HIN-domain of the AIM2 receptor, releasing the receptor from its autoinhibitory state. This allows the AIM2 receptor to oligomerize and initiate inflammasome activation. Abbreviations: CCH, chronic cerebral hypoperfusion; ROS, reactive oxygen species; ATP, adenosine triphosphate; P2X4, P2X purinoceptor 4; P2X7, P2X purinoceptor 7; NLR, nucleotide-binding oligomerization domain-like receptor; NLRP1, NLR family pyrin domain containing 1; NLRP3, NLR family pyrin domain containing 3; cAMP, cyclic adenosine monophosphate; TRPM2, transient receptor potential melastatin 2; TXNIP, thioredoxin-interacting protein; TRX, thioredoxin; AIM2, absent in melanoma 2; HIN200, hematopoietic interferon-inducible nuclear proteins; dsDNA, double-stranded DNA; RAGE, receptor for advanced glycation end-products

During the pathogenesis of VCI, a multitude of stress signals and DAMPs are produced in the cytosol, released and detected by various PRRs, resulting in their eventual activation and formation of inflammasome complexes [194, 200]. This section discusses stimuli that potentially activate the NLRP1, NLRP3, NAIP-NLRC4 and AIM2 inflammasome receptors during CCH (Fig. 4).

The exact function of NLRP1 in innate immunity is not yet fully elucidated, but it is believed to be activated via autolytic proteolysis within the FIIND domain in response to severe bacterial infections [220]. NLRP1 levels are upregulated in ischemic conditions, and although the responsible activation signals are unclear, they are likely to be from aberrations in the cellular microenvironment such as depletion of intracellular ATP and reduction of intracellular K^+^ levels arising from K^+^ efflux during bioenergetic impairment [38, 212, 230]. The NLRP3 receptor can be activated by a multitude of intracellular signals including decreased K^+^, increased Ca^2+^ and oxidative stress during CCH [212, 231, 232]. Given that NLRP3 can respond to a diverse range of signals, it is likely to be responding to a common cellular event caused by these activators rather than directly to the activators themselves. Moreover, increased levels of hyaluronic acid was observed in the cerebrospinal fluid of VaD patients that appeared to serve as a potential NLRP3 stimuli [233].

While no study to date has directly investigated the effect of CCH to cause bioenergetic deficits, blood flow reduction during ischemia is known to induce K^+^ efflux [46, 234]. This is a possible mechanism by which CCH activates the NLRP inflammasome receptors. Lower cerebral blood flow reduces ATP production and impairs ATP-dependent transporters such as the Na^+^/K^+^-ATPase pump leading to K^+^ accumulation in the extracellular space during ischemia [46, 234]. Alternatively, ATP released by damaged cells can bind to the P2X purinoceptor 4 (P2X4) and P2X purinoceptor 7 (P2X7) on neighboring cells, leading to opening of the ligand-gated ion channel and K^+^ efflux. In addition, K^+^ can be passively released into the extracellular environment due to increased permeability of the plasma membrane from damaged cells [38, 235]. Elevated levels of extracellular K^+^ can activate Pannexin-1 channels on the plasma membrane through a mechanism independent of the membrane potential, to further promote release of ATP, creating a positive feedback loop for K^+^ efflux [38, 236, 237]. K^+^ efflux has been identified as a key step for NLRP1 and NLRP3 inflammasome activation, although there is no clear understanding of the precise mechanism linking K^+^ efflux and NLRP receptor activation [232, 238]. However, a recent study showed that low intracellular K^+^ concentrations can open the inactive structure of NLRP3 by altering a domain in between the PYD and NACHT domains, resulting in a stable structure that promotes the functional oligomerization of NLRP3 into active oligomers [239] (Fig. 4).

Other than K^+^ efflux, a reduced intracellular cyclic adenosine monophosphate (cAMP) concentration during CCH may activate the NLRP3 inflammasome [235, 240]. Binding of cAMP to the NLRP3 receptor inhibits its ability for inflammasome assembly. When cAMP is reduced upon CCH, NLRP3 activation is promoted [231, 235, 240]. While the underlying cause of cAMP reduction is unknown, evidence from mechanistic studies from ischemic models suggest an involvement of Ca^2+^. When Ca^2+^ is released by damaged cells, it activates G-protein coupled calcium-sensing receptors (CaSRs) on the plasma membrane, allowing it to interact with Gαi and inhibit adenylate cyclase, reducing the conversion of ATP to cAMP in neighboring cells [38, 231, 235]. The involvement of Ca^2+^ in mediating NLRP3 inflammasome activation is highly plausible as the Ca^2+^-permeable channel, transient receptor potential melastatin 2 (TRPM2), plays a significant role in regulating the production of IL-1β upon CCH [70]. One possible explanation for this is that it allows influx of Ca^2+^, which activates protein kinase R (PKR) in the cytoplasm. Upon activation, PKR can phosphorylate NLRP1 or NLRP3 receptors for inflammasome activation, leading to production of IL-1β [38, 241, 242] (Fig. 4).

Numerous lines of evidence have shown that NLRP3 inflammasome activation is closely linked to increased levels of ROS in neurological diseases [243, 244]. Several studies have already demonstrated a close association between ROS, thioredoxin-interacting protein (TXNIP) and NLRP3 [243–245]. The generation of ROS facilitates the uncoupling of TXNIP from thioredoxin (TRX), allowing TXNIP to bind to the NLRP3 receptor for inflammasome activation [244–246]. Due to increased oxidative stress observed in CCH mouse models of VCI [39, 55, 247], one study demonstrated that decreased TXNIP-associated oxidative stress was associated with reduced NLRP3 and IL-1β expression, in conjunction with better cognitive performance [248]. Another major source of ROS is from the mitochondria as increased hydrogen peroxide production induced oxidative stress that were observed in rodent models of CCH [39] (Fig. 4).

NAIP-NLRC4 is generally activated by pathogenic bacteria, with NAIP as a direct receptor to these bacterial signals [249] However, activation of NAIP-NLRC4 has been shown to be associated with lysophosphatidylcholine (LPC), which are lipids arising from the hydrolytic activity of phospholipase A2 (PLA_2_) under stressed conditions [250, 251]. Although LPC has been found to activate NLRC4 and NLRP3 inflammasomes in neuroinflammatory disease mouse models [250, 252], it is unlikely to be involved in VCI as the levels of LPC do not differ significantly upon CCH [253]. Nucleotide-derived metabolites, including adenine and N4-acetylcytidine (N4A), can both prime and activate the NAIP-NLRC4 inflammasome [254], and could be relevant given that oxidative DNA damage has been described in VCI patients [60]. However, no evidence to date have investigated the effect of CCH on nucleotide-derived metabolites.

AIM2 is activated by the binding of dsDNA to the HIN domain, thereby removing the autoinhibitory effect of the intramolecular interaction between the PYD-HIN domains. This ligand binding is achieved via electrostatic attractions between the positively-charged HIN domain and negatively-charged dsDNA [218, 223, 255]. The dsDNA is required to be at least 80 base pairs in length and is conventionally from viral or bacterial origins [256]. Given the sterile nature of VCI, it is likely that AIM2 is activated by host dsDNA instead because ischemic conditions produce anoxic depolarization and release of mitochondrial DNA into the cytosol due to ATP-induced mitochondrial apoptosis. Coupled with the extracellular release of nuclear and mitochondrial DNA by injured neurons and glial cells, this provides the appropriate activation signals in the form of dsDNA to AIM2 [257, 258]. During CCH, DNA fragmentation has been observed to occur in astrocytes and oligodendrocytes [130, 259]. Furthermore, it was recently shown that CCH increases plasma levels of double-stranded DNA, and induces AIM2 inflammasome-mediated neuropathology and cognitive impairment in a mouse model of VaD [71]. Moreover, CCH-induced receptor for advanced glycation end-products (RAGE) upregulation can also promote DNA uptake into the cell via the action of endosomes, hence providing a mechanism through which extracellular DNA can interact with cytosolic AIM2 [260, 261] (Fig. 4).

### Formation of the Inflammasome complex

**Fig. 5:**
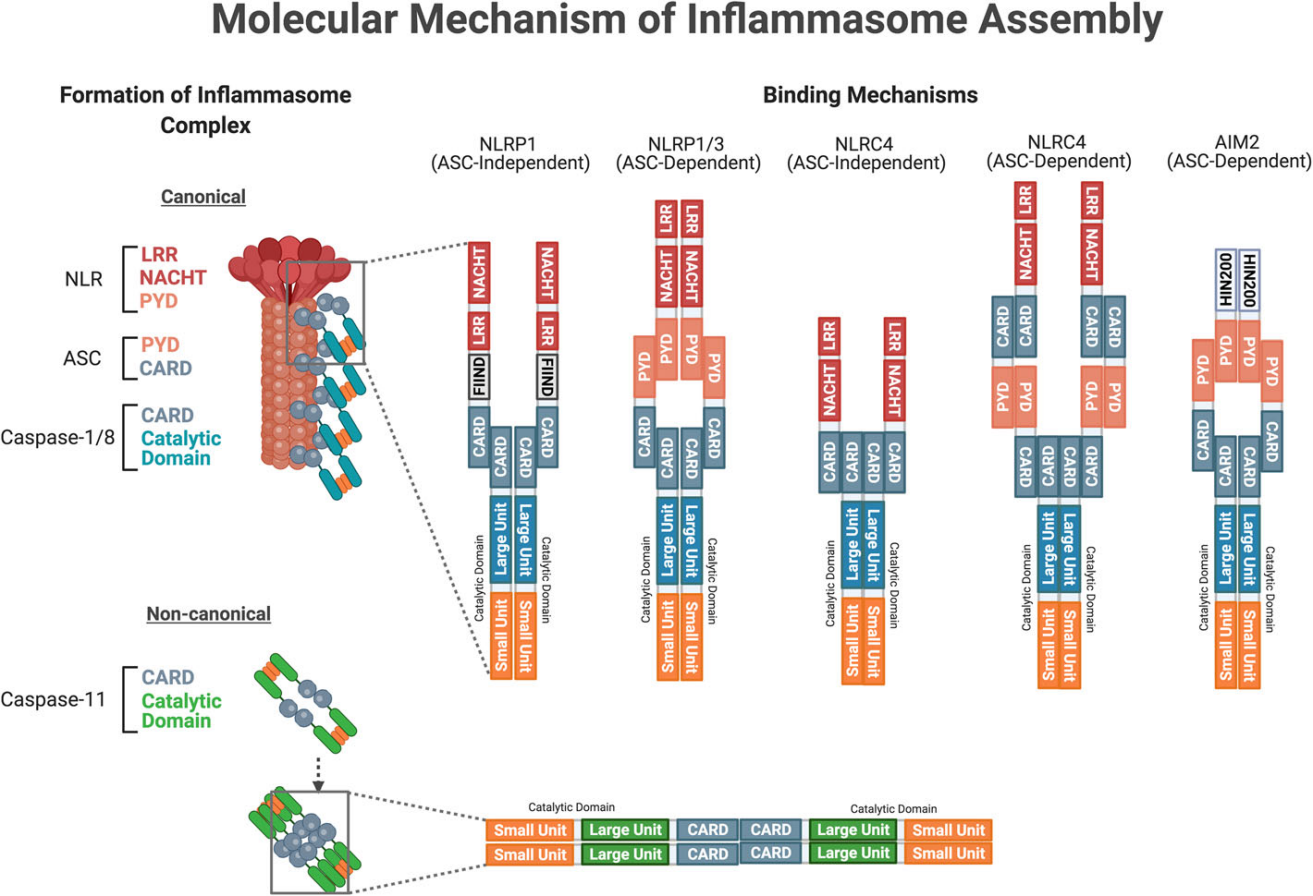
A schematic diagram illustrating the assembly of the canonical and non-canonical inflammasome complexes. The formation of the canonical inflammasome complex requires the activation of inflammasome receptors from the second signal. As the LRR inhibitory unit unfolds from the NACHT domain, the receptors are in an “open” structure for homotypic oligomerization through their NACHT domain. Subsequently, the PYD domain recruits the adaptor protein via the PYD domain on ASC. As numerous ASC adaptor proteins comes together, they will form a filamentous structure with their CARD domain exposed. Consequently, effector proteins with their CARD domain can bind to the filamentous structure via CARD-CARD interactions. Such protein aggregation triggers proximity-induced activation of total caspase-1, and − 8, leading to cleavage of the inter-domain linker between the large and small units, producing active cleaved caspase-1 and -8. The above mentioned ASC-dependent binding mechanism generally applies to all inflammasome complexes. However, NLRP1 can form an inflammasome complex without the adaptor ASC. Using its C-terminal CARD domain, NLRP1 binds to the effector protein via the CARD-CARD domain. NLRC4 can also adopt the same ASC-independent binding mechanisms with the CARD domain on the receptor. In the non-canonical inflammasome pathway, caspase-11 can undergo homo-oligomerization in the absence of a receptor and adaptor protein component via the CARD-CARD domain interaction. Similar to the activation of total caspase-1 and -8, cleaved caspase-11 is produced upon proximity-induced activation. Abbreviations: NLR, nucleotide-binding oligomerization domain-like receptor; NACHT, NAIP (neuronal apoptosis inhibitor protein) C2TA (class 2 transcription activator, of the MHC) HET-E (heterokaryon incompatibility) and TP1 (telomerase-associated protein 1); LRR, leucine-rich repeat; PYD, pyrin domain; CARD, caspase recruitment domain; NLRP1, NLR family pyrin domain containing 1; NLRP3, NLR family pyrin domain containing 3; NLRC4, NLR family CARD domain-containing protein 4; AIM2, absent in melanoma 2; HIN200, hematopoietic interferon-inducible nuclear proteins with a 200-amino-acid repeat; dsDNA, double-stranded DNA; ASC, apoptosis-associated speck-like protein containing a CARD; DED, death effector domain

In general, stimulation of the inflammasome receptor causes the LRR inhibitory unit to unfold from the NACHT domain [38, 216]. Consequently, the inflammasome receptor reconfigures into an “open” structure to allow homo-oligomerization with neighbouring inflammasome receptors. As more inflammasome receptors converge, they form a macro-molecular platform with their N-terminal PYD domains pointing towards each other [224, 262]. A PYD domain attracts other PYD domains via homotypic interactions. One of them is the adaptor protein, ASC, which facilitates inflammasome complex formation by binding to the PYD domain of the inflammasome receptor platform using its N-terminal PYD domain through homo-oligomerization [38, 224]. Many ASC proteins will come together during this process, forming a filamentous structure from the inflammasome receptor platform. These filamentous macromolecular aggregates are known as ASC specks [263]. As such, the CARD domain on the C-terminal of ASC is made available to bind with full-length caspases. These caspases bind with their N-terminal CARD domain to the ASC specks via CARD-CARD domain interactions [217, 263], and therefore directly interact with CARD domains on inflammasome receptors in the absence of the ASC adaptor. NLRP1 and NLRC4 receptors can mediate ASC-independent inflammasome activation [249, 264]. The structural formation of each major inflammasome complex is explained below (Fig. 5).

Following stimulation of the NLRP1 inflammasome receptor, the FIIND domain undergoes autolytic proteolysis to facilitate activation and oligomerization to form the inflammasome core [220]. NLRP1 oligomers appear to interact with ASC via homotypic PYD-PYD interactions [220]. After ASC speck formation, the ASC speck can recruit multiple effector inflammatory caspases via homotypic CARD-CARD interactions [219]. Despite being able to mediate ASC-independent NLRP1 inflammasome activation, NLRP1 activity level is higher in the presence of an ASC speck [220] (Fig. 5). Prior to NLRP3 activation, priming must occur in the form of increased expression and de-ubiquitination of NLRP3 [199, 201]. Assembly of the NLRP3 inflammasome also requires ASC to be linearly ubiquitinated [265]. The NLRP3 receptor binds to ASC via homotypic PYD-PYD interactions, and through the resulting ASC speck recruits effector inflammatory caspases via homotypic CARD-CARD interactions [221, 224] (Fig. 5). Upon binding of NAIP to the ligand, a single NAIP oligomerizes with multiple NLRC4 receptors. Phosphorylation of a single evolutionarily conserved serine 533 residue in NLRC4 is necessary for the assembly of the NAIP-NLRC4 inflammasome [266]. NAIP-NLRC4 then interacts with ASC likely through homotypic CARD-CARD interactions, given the absence of a PYD domain in the NAIP-NLRC4 receptor. ASC speck formation ensues and is accompanied by recruitment of multiple effector inflammatory caspases through homotypic CARD-CARD interactions. NLRC4, however, possesses an innate CARD domain, and so can recruit the effector inflammatory caspases independent of ASC, although the presence of ASC can enhance the assembly of the NAIP-NLRC4 inflammasome complex [222, 249, 263] (Fig. 5). The PYD domain of AIM2 possesses an intrinsic tendency to self-aggregate, resulting in homo-oligomerization of several AIM2 receptors after activation via dsDNA binding to the HIN domain. The AIM2 receptor complex then recruits ASC concomitant with ASC speck formation via homotypic PYD-PYD interactions. AIM2 recruits multiple effector inflammatory caspases via homotypic CARD-CARD interactions in an ASC-dependent manner, due to the absence of CARD in the AIM2 receptor [255, 267] (Fig. 5).

The molecular assembly of different inflammasome complexes can vary between inflammasome receptors in the canonical pathway. The ultimate purpose of inflammasome assembly is to bring together the effector caspases in close proximity. Increasing the local concentration of effector caspase-1 around the complex facilitates dimerization of caspase-1 monomers, and enables autoproteolytic activation [255, 267]. Subsequently, a transient activity hub of cleaved caspase-1 p33/p10 is produced for substrate catalysis. Different cell types and inflammasome sizes influence the kinetics of p33/p10 processing. Therefore, the final product of cleaved caspase-1 p20/p10 serves as a classic hallmark of caspase-1 activation as it indicates termination of protease activity [226, 268] (Figs. 2, 3 & 5). Unlike caspase-1, which binds to the inflammasome complex via CARD-CARD interactions, caspase-8 relies on its DED domain to carry out heterotypic interactions with the adaptor ASC. This is possibly due to the similarities with its self-association between DEDs and PYDs domains. As such, caspase-8 is similarly able to undergo proximity-induced activation, producing cleaved caspase-8 for substrate processing [224, 269, 270]. Nonetheless, caspase-8 is capable of cleaving inflammasome substrates (IL-1β) directly via the complex formation of caspase recruitment domain-containing protein-9 (CARD9), B-cell lymphoma/leukemia-10 (BCL10), Mucosa-associated lymphoid tissue lymphoma translocation protein-1 (MALT1), ASC and caspase-8. Caspase-8 can also interact with receptor-interacting serine/threonine kinase 1 (RIPK1) directly cleaving caspase-1 leading to caspase-1 activation [205, 206].

Organization of the canonical inflammasome complex usually involves a receptor, adaptor and effector caspases-1 and -8. However, in the non-canonical inflammasome pathway, caspase-11 can undergo homo-oligomerization in the absence of a receptor and adaptor protein component. Upon binding of a stimuli such as oxPAPC or LPS to the N-terminal CARD domain on caspase-11, the CARD domain interacts with the same domain on another caspase-11 to form a dimer [271, 272]. The dimer then interacts with others to form a homo-tetramer, which then oligomerizes to induces auto-proteolysis. As caspase-11 undergoes self-cleavage, it releases the pro-domain from the catalytic domain of caspase-11, producing biologically active cleaved caspase-11 [271, 272] (Figs. 2, 3 & 5).

### Impact of the Inflammasome signaling pathway

Upon the activation of the inflammasome signaling pathway, the mature cytokines IL-1β and IL-18, are produced leading to the downstream inflammatory response. Simultaneously, these effector proteins also initiate a wide variety of cell death pathways such as apoptosis, pyroptosis and secondary necrosis [38] (Fig. 2).

#### Proinflammatory effect of IL-1β and IL-18

The proinflammatory cytokines, IL-1β and IL-18, are both transcriptionally upregulated during inflammasome priming [199]. They are produced in the form of precursor cytosolic proteins activated by canonical cleaved caspase-1 and -8, while cleaved caspase-11 aids in the release of these cytokines into the extracellular environment by inducing gasdermin pores [214, 268] (Fig. 2).

Following their production and release into the extracellular environment, mature IL-1β and IL-18 serve as ligands towards the IL-1R1 and IL-18R on the plasma membrane to facilitate an autocrine or paracrine effect. Consequently, this activates the NF-κB and MAPK(s) pathways to upregulate gene expression of several types of inflammatory mediators [38, 198, 273, 274]. The first type is proinflammatory cytokines (i.e. TNF, IL-1β, IL-6 and IL-8) that further propagate the inflammatory signal. The second is chemotactic cytokines (e.g. CXC-chemokine ligand 8 and CX3C-chemokine ligand 1), which attract peripheral immune cells such as neutrophils and macrophages to the damaged region. The third type is adhesion molecules (e.g. E-selectin and ICAM-1) that facilitate leukocyte infiltration into the brain parenchyma [275, 276]. These recruited peripheral immune cells can initiate a similar inflammatory response, and contribute to the pool of inflammatory mediators released from microglia and astrocytes [277, 278].

#### Effect of Caspase-1, − 8 and − 11 on cell death pathways

Apoptosis is a form of programmed cell death that encompasses the intrinsic and extrinsic apoptotic pathways. Activation of both pathways ultimately converge to activate executioner caspase-3, leading to apoptosis characterized by membrane blebbing, cell shrinkage, nuclear fragmentation, chromatin condensation, chromosomal DNA fragmentation and global mRNA decay [279, 280]. Inflammasomes induce apoptosis via recruitment and activation of caspase-8. Moreover, caspase-1 is also implicated in apoptosis through caspase-1 mediated cleavage of caspase-7 or caspase-3, both of which are involved in the execution of apoptosis [207, 281]. Caspase-11 can also activate caspase-3 to induce apoptosis in a caspase-1 independent manner [282]. Besides its apoptotic function, cleaved caspase-3 can induce secondary necrosis by cleaving the hallmark protein, gasdermin E to produce the N-terminal fragment (NT-GSDME). Secondary necrosis involves the oligomerization of these N-terminal fragments, leading to pore formation in the mitochondrial membrane and cellular surface [283] (Fig. 2), thus allowing cytochrome c to be released into the cytosol, further promoting apoptotic cell death [284].

Pyroptosis is an inflammasome-driven programmed cell death pathway that is initiated by the activation of caspase-1 and -11 resulting in cellular lysis [208, 210, 211]. Gasdermin D is an integral pyroptotic protein comprised of an N-terminal domain capable of forming a pore in the plasma membrane, and a C-terminal domain which represses the activity of the N-terminal gasdermin D domain (Fig. 2). Proteolytic cleavage of gasdermin D by either caspase-1 or − 11 relieves the inhibitory effect of the C-terminal gasdermin D domain on the N-terminal gasdermin D domain, allowing for translocation of N-terminal gasdermin D to the plasma membrane where it integrates and oligomerizes to form a gasdermin D pore [208, 210, 211]. Pore formation results in K^+^ efflux and influx of both Na^+^ and water molecules, resulting in cell swelling and lysis if there is a sufficient number of gasdermin D pores [208, 210, 211]. Pyroptosis is regarded as a proinflammatory form of cell death whereby proinflammatory cytokines such as IL-1β and IL-18 are usually restricted to the cytosol due to the lack of a secretory system but, along with other DAMPs and inflammatory mediators, they can be released into the extracellular environment after gasdermin pore formation [208, 210, 211]. Recently, caspase-8 was also demonstrated to cleave gasdermin D to induce pyroptosis [209]. In the absence of caspase-1 or gasdermin D, NAIP-NLRC4 can recruit and activate caspase-8 to induce a pyroptotic-like cell death involving the formation of membrane pores in a gasdermin D-independent manner [285].

### Inflammasomes in Glial activation

As mentioned, microglia and astrocytes are key players in mediating neuroinflammation and tissue damage. Microglial cells are equipped with different PRRs that screen the microenvironment in the resting state [286, 287]. In an RNA-sequencing transcriptome study using mouse brain, gene expression of NLRP1, NLRP3, NLRC4 and AIM2 in the microglia was highest among all brain cell types. Moreover, microglial expression of caspase-1 and IL-1β was around four and thirty fold higher than neurons, respectively [288]. This primes microglia for rapid activation when CCH induces ionic imbalance, oxidative stress and inflammation. Canonical classification of activated microglia is broadly described as either proinflammatory M1-like or anti-inflammatory M2-like phenotypes. M1-polarised microglia produce proinflammatory cytokines such as IL-1β and TNF-α that serve to activate more microglia cells in a paracrine manner [289]. However, emerging transcriptomic studies revealed more disease-associated subtypes of microglia such as Keratan sulfate proteoglycan (KSPG)-microglia (associated with amyotrophic lateral sclerosis), highly active “dark microglia” which interact with blood vessels and synapses (associated with Alzheimer’s disease) and CD11c-microglia which interact with oligodendrocytes (associated with demyelination) [290]. Based on current evidence, microglia are likely to promote inflammatory and non-inflammatory responses via an involvement of NLRP3 and AIM2 inflammasomes during VCI [71, 291]. In fact, a study showed reduced proinflammatory microglia signatures in the hippocampus of mice with AIM2 deficiency upon CCH [71]. Attenuating NLRP3 inflammasome activity via pharmacological inhibitors during CCH also reduces microglial overactivation, possibly due to lower production of ROS during drug treatment [291].

Astrocytes are another immune effector cell type in the brain. Similar to microglial polarization states, activated astrocytes exist in two phenotypes based on traditional understanding: neurotoxic A1 and neuroprotective A2 astrocytes [292, 293]. Beyond this binary classification, reactive astrocytes were found to be more heterogeneous in terms of their morphology, locality, cellular interaction, and molecular expression. Therefore, the status of reactive astrocytes varies in a context-, time- and stimulus-specific manner [294]. In the context of neurodegenerative diseases, reactive astrocytes are consistently being identified with hypertrophied morphology and reduced expression of essential ion and neurotransmitter channels and receptors such as ATP-sensitive inward rectifier potassium channel 10 (Kir4.1), glial glutamate transporter 1 (GLT1), and increased expression of the glial fibrillary acidic protein (GFAP). This state of reactive astrocytes is commonly observed in A1 astrocytes that release proinflammatory and toxic mediators [294–297]. Both A1 and A2 phenotypes were present in conjunction with inflammasome activation following CCH.

Astrocytes have been shown to express NLRP1, NLRP3, AIM2 and NLRC4 receptors under different conditions. However, genetic deletion of the AIM2 receptor did not affect the expression of GFAP in either the cortex or hippocampus of mice during CCH, suggesting potential involvement of other inflammasome receptors [71, 257, 298, 299]. We recently collected unpublished data that the inflammasome signaling pathway is activated in fibrous and protoplasmic astrocytes under in vitro ischemic conditions, resulting in mature cytokine production, and apoptotic and pyroptotic cell death. Hence, we postulate that inflammasome activation may serve as a signal for astrocyte polarization.

### Inflammasomes in BBB dysfunction

BBB disruption can arise from neuroinflammation, primarily through degradation of various tight junction proteins (TJPs). Matrix metalloproteinases (MMPs) released by glial cells can break down the extracellular matrix [300] and have been implicated in the disruption of the BBB under ischemic conditions [104]. MMP-2 and MMP-9 are known to be involved in BBB disruption and primarily localized to ischemic regions of astrocytic foot processes, during which TJPs undergo degradation over time [301]. Inflammasome signaling contributes to BBB dysfunction via the action of IL-1β, which can upregulate the expression and release of MMPs from glial cells [302, 303]. Application of exogenous IL-1β elicits BBB dysfunction in rat and human brain microvascular endothelium [94, 304]. In addition, both IL-1β and IL-18 can upregulate expression of various chemokines in the extracellular space and cell adhesion molecules on the endothelium [276, 305]. Cell adhesion molecules, such as ICAM-1 and VCAM, attract and facilitate immune cell infiltration into the brain during neuroinflammation. These peripheral cells can also release active MMP-9, further exacerbating BBB dysfunction [306, 307]. A recent study showed that an interleukin-1 receptor antagonist preserved BBB integrity, attenuated changes in expression and localization of TJPs and MMPs in a rat model of ischemia-reperfusion [308]. Inhibition of caspase-1 enzymatic activity in a similar model further revealed caspase-1 to induce BBB dysfunction through the activation of pyroptosis [309]. Therefore, a number of studies support a critical role of inflammasomes in BBB disruption, especially following disruption to CBF.

As an essential component of the BBB, the endothelial cell expresses a wide range of inflammasome receptors such as NLRP1, NLRP3 and NLRC4 [310]. Studies showed endothelial NLRP3 in modulating the BBB during different disease conditions, but evidence for CCH is still lacking [311–313]. NLRP3 may mediate BBB dysfunction for CCH based on in vitro and in vivo evidence from studies focusing on ischemic brain injury. Pharmacological inhibition of NLRP3 inflammasome activity has been shown to attenuate cerebral ischemia-induced BBB dysfunction by reducing its permeability and upregulating TJPs [313]. In addition, the study confirmed that reduced NLRP3 activity in endothelial cells increased expression of TJPs, cell viability and reduced barrier leakage [313]. This mechanism observed may explain the BBB dysfunction that is observed during CCH.

Pericytes also express the NLRP3 receptor along with other NLRP and NLRC inflammasome receptors [314]. Upon stimulation of pericytes by oxidative stress and proinflammatory mediators, in vitro cerebral pericytes demonstrated upregulation of NLRP3 and NLRC4 mRNA expression [314]. Despite an increase in inflammasome receptor mRNA expression, activation of the inflammasome complex was not detected in cerebral pericytes following exposure with a wide range of DAMPs [314]. However, as pericytes are increasingly implicated in BBB dysfunction during CCH [91, 100, 101], additional studies are needed to establish the role of inflammasomes in pericytes during VCI.

### Inflammasome mediated cell death and neuronal loss

Inflammation is closely linked to cell death at the molecular level. The cell death pathway can be triggered by various proinflammatory mediators [315]. As DAMPs initiate the inflammasome signaling pathway via the NLR family and interferon-inducible protein, it activates key effector proteins such as caspase-1 and caspase-8 to cleave caspase-3, resulting in apoptosis and secondary necrosis [206, 283]. Together with the non-canonical caspase-11, these effector proteins catalyze pyroptosis via cleavage of gasdermin-D (GSDMD) [203, 208, 210]. Inhibition of caspase-1 reduces inflammasome activation and cell death in primary cortical neurons and murine microglial cells subjected to ischemia-like conditions [200, 203]. The NLRP3 inhibitor, MCC950, decreases apoptotic cell death and brain infarct size in a mouse model of ischemic stroke [316]. In a mouse model of VCI, AIM2 knockout mice expressed reduced inflammasome activity as well as apoptotic and pyroptotic cell death in neurons and microglia in the cortex and hippocampus. Similarly, a higher neuronal count was observed in the CA2 and CA3 area of the hippocampus in AIM2 knockout mice during CCH [71]. Therefore, the AIM2 inflammasome is involved in CCH-induced neuroinflammation by mediating cell death and neuronal loss during VCI disease progression.

### Role of Inflammasomes in demyelination and WMLs

Numerous studies have indicated a close association of inflammasome activity with the formation of WMLs that often occur together with activated microglia [77, 203, 317]. As mentioned, activated microglia release numerous inflammatory mediators that contribute to demyelination, including IL-1β and IL-18 that are produced during inflammasome activation. IL-1β was shown to impede oligodendrocyte migration and white matter repair in mouse models of VCI [69]. Conversely, reduced secretion of IL-1β preserved myelin integrity and attenuated the formation of WMLs under CCH [71]. Anti-inflammatory pharmaceutical interventions and transgenic animal models have attenuated WML formation via suppressing microglial activation, and preventing caspase-1 and IL-1β production [70, 318, 319]. Other than in microglia, inflammasomes are also activated during CCH in oligodendrocytes. Our recent study found increased levels of cleaved caspase-1 in oligodendrocytes of mice after 30 days of BCAS [71]. Similar observations of inflammasome-mediated apoptotic and pyroptotic cell death markers were also found within oligodendrocytes. The evidence suggests that the inflammasome signaling pathway likely plays a causative role upstream of CCH-induced WML formation.

## Evidence of inflammasome activity in VCI in humans

An involvement of inflammasome activation and cytokine production is related to the risk factors that drives the early disease state of VCI. In tissue samples from patients with atherosclerosis, IL-1β is detected within endothelial cells of plaque microvessels [320]. Similarly, high levels of NLRP3, ASC, caspase-1, IL-1β, and IL-18 mRNA expression was observed in carotid artery plaques of patients with cerebrovascular disease [321]. Studies of polymorphisms of IL-1β among small vessel disease patients, there was a higher frequency of the IL-1β allele in comparison to controls [322], suggesting that elevated levels of IL-1β may contribute to the progression of small vessel disease leading to VCI. Genetic investigation of coronary artery disease patients also found that patients carrying the G allele of NLRP3 rs10754558 had more severe coronary artery stenosis and higher levels of serum IL-1β [323]. With the G allele of NLRP3 rs10754558 enhancing the mRNA stability of NLRP3, it increases the mRNA expression of this inflammasome receptor, potentially contributing to the pathophysiology of coronary artery stenosis [323, 324]. Besides chronic vascular conditions, inflammasome activity has also been identified in VCI-associated acute conditions such as stroke [38]. By preventing binding of IL-1β with its receptor, a clinical trial showed that acute stroke patients experienced a better outcome following the administration of an interleukin-1 receptor antagonist (i.e. anakinra) [274]. This was shown by a reduction in inflammation due to lower levels of neutrophils and C-reactive proteins in the systemic circulation following a cerebral infarction. Among patients with cortical infarction, the use of an interleukin-1 receptor antagonist significantly reduced long-term disability after three months following cerebral ischemia; demonstrating the beneficial effect of reducing inflammasome activation and IL-1β secretion in cerebral vascular disease [274]. Similarly, IL-18 was shown to be more commonly associated with peripheral arterial occlusive disease, and cerebrovascular events [325], whereby an elevated level of IL-18 was shown to be evident in the plasma of acute coronary syndrome patients [326].

As the disease progresses to late-stage VCI, the actions of inflammasomes persist. In post-mortem samples from VaD patients, increases in the concentration of IL-1β was observed in the frontal cortex and hippocampus [66, 193]. Through cytokine profiling, studies of serum and plasma of VaD patients showed levels of IL-1β to be higher than in healthy controls. The relative increase in IL-1β was higher than other proinflammatory cytokines such as TNF-α and IL-6 [64, 65]. Nonetheless, some studies face challenges in detecting IL-1β in their patient samples [183, 184, 187]. For example, Mulugeta et al. did not detect any IL-1β in their patient brain tissues using ELISA kits [184]. Paganelli et al. found detectable levels of IL-1β only in 13% of all their serum samples through the same method [187]. Thus there is substantial variability in the interpretations of IL-1β in VCI because the levels present are near the limits of precise measurement by ELISA.

In addition to the presence of inflammasome-mediated proinflammatory cytokine production, immunostaining of inflammasome receptors, NLRP3 and AIM2, was greater in white matter lesions of patients with cerebral infarction [317]. Together these studies provide evidence of inflammasome activation in a severe state of VCI, highlighting the prominence of inflammasome signaling in the disease progression of VCI.

## Conclusions

This review illustrates the mechanisms of inflammasome-mediated neuroinflammation under CCH in VCI. By providing direct evidence of inflammasome activation in VCI animal models and patients, we suggest that the critical involvement of the inflammasome signaling pathway in the pathogenesis of VCI may be mediated through CCH [64–66, 71, 118, 119, 193, 317]. Most importantly, inflammasome-mediated inflammatory mechanisms are early events that persist till the late stage of VCI; this sheds light on the inflammasome signaling pathway as a potential therapeutic target [64–66, 71, 118, 119, 193, 317]. By reducing inflammasome activity levels, we can attenuate its influence on various pathogenic cellular mechanisms and structural damage observed during the disease progression of VCI [119, 291]. There are clinically approved therapeutic agents that can effectively target the actions of IL-1β: anakinra, canakinumab, and rilonacept. Moreover, agents that neutralize the effects of IL-18, Tadekinig alfa and GSK1070806 are also undergoing clinical trials [327]. It is essential to keep in mind that the inflammasome signaling pathway is a significant player within our innate immune system and physiologically guards against infectious agents [328]. Hence, there is a challenge to maintain an equilibrium in reducing inflammasome activity while maintaining our defense against infectious agents. Thus, the search for therapeutic interventions that selectively target specific types of inflammasome complexes may offer a greater safety and efficacy profile in the long term, especially for chronic diseases such as VCI.

## Data Availability

Not applicable.

## Abbreviations

ACAS: Asymmetric Common Carotid Artery Stenosis
AD: Alzheimer’s Disease
AIM2: Absent in Melanoma 2
AP-1: Activator Protein-1
ASC: Apoptosis-associated Speck-like protein containing CARD
ATP: Adenosine Triphosphate
BBB: Blood-Brain Barrier
BCAS: Bilateral Common Carotid Artery Stenosis
Bcl-2: B-cell Lymphoma-2
C5a: Complement Factor 5a
CAA: Cerebral Amyloid Angiopathy
CADASIL: Cerebral Autosomal Dominant Arteriopathy with Subcortical Infarcts and Leukoencephalopathy
cAMP: Cyclic Adenosine Monophosphate
CaSRs: Calcium-sensing receptors
CARD: Caspase Activation and Recruitment Domain
CBF: Cerebral Blood Flow
CCH: Chronic Cerebral Hypoperfusion
CIITA: MHC class II Transcription Activator
CRP: C-reactive protein
DAMPs: Damage-Associated Molecular Patterns
DED: Death Effector Domains
dsDNA: Double-stranded DNA
FIIND: Function-to-Find Domain
GFAP: Glial Fibrillary Acidic Protein
GLT1: Glial Glutamate Transporter 1
GSDMD: Gasdermin D
GSDME: Gasdermin E
H_2_O_2_: Hydrogen Peroxide
HIF-1: Hypoxia-Inducible Factor-1
HRP: Horseradish Peroxidase
ICAM: Intercellular Adhesion Molecule
IFN: Type I interferon
IL-1β: Interleukin-1β
IL-6: Interleukin-6
Kir4.1: ATP-sensitive Inward Rectifier Potassium Channel 10
LPC: Lysophosphatidylcholine
LPS: Lipopolysaccharides
LRR: Leucine-Rich Repeat
MALT1: Mucosa-Associated Lymphoid tissue lymphoma Translocation protein-1
MAPKs: Mitogen-Activated Protein Kinases
MID: Multi-Infarct Dementia
MMPs: Matrix Metalloproteinases
MPB: Myelin Basic Protein
mtROS: Mitochondrial ROS
NADPH: Nicotinamide Adenine Dinucleotide Phosphate
NAIP: NLR Apoptosis Inhibitory Protein
NAIP: NLR Apoptosis Inhibitory Protein
NF-κB: Nuclear Factor Kappa B
NLR: Nod-Like Receptor
NLR: Nucleotide-binding ligomerization domain-like receptor
NLRC4: NLR-CARD Containing 4
NT-GSDMD: N-terminal fragment GSDMD
NT-GSDME: N-terminal fragment GSDME
OH^-^: Hydroxyl Radical
oxPAPC: Oxidized 1-palmitoyl-2-arachidonyl-*sn*-glycero-3-phosphorylcholine
P2X4: P2X purinoceptor 4
P2X7: P2X purinoceptor 7
PAMPs: Pathogen-Associated Molecular Patterns
PLA_2_: Phospholipase A2
PKR: Protein kinase R
PRRs: Pattern Recognition Receptors
PYD: Pyrin
PYHIN: Nuclear localization (HIN) domain-containing
RAGE: Receptor for Advanced Glycation End-products
RIPK1: Receptor-Interacting serine/threonine Kinase 1
ROS: Reactive Oxygen Species
SVD: Small Vessel Disease
TJPs: Tight Junction Proteins
TLRs: Toll-Like Receptors
TNF: Tumor Necrosis Factor
TP1: Telomerase-associated Protein
TRPM2: Transient receptor potential melastatin 2
TXNIP: Thioredoxin-interacting protein
TRX: Thioredoxin
UCCAO: Unilateral Common Carotid Artery Occlusion
VaD: Vascular Dementia
VCAM: Vascular Cell Adhesion Molecule
VCI: Vascular Cognitive Impairment
WML: White Matter Lesion
